# Revealing signaling pathway deregulation by using gene expression signatures and regulatory motif analysis

**DOI:** 10.1186/gb-2007-8-5-r77

**Published:** 2007-05-11

**Authors:** Yingchun Liu, Markus Ringnér

**Affiliations:** 1 Computational Biology and Biological Physics, Department of Theoretical Physics, Lund University, SE-221 85, Sweden; 2 Division of Oncology, Department of Clinical Sciences, Lund University, SE-221 85, Sweden

## Abstract

Gene expression signatures consisting of tens to hundreds of genes have been found to be informative for different biological states. Recently, many computational methods have been proposed for biological interpretation of such signatures. However, there is a lack of methods for identifying cell signaling pathways whose deregulation results in an observed expression signature. We present a strategy for identifying such signaling pathways and evaluate the strategy using six human and mouse gene expression signatures.

## Abstract 2

A strategy for identifying cell signaling pathways whose deregulation result in an observed expression signature is presented.

## Background

Genetic aberrations and variations in cellular processes are usually reflected in the expression levels of many genes. Hence, such alterations can potentially be characterized by their gene expression profiles. Gene expression profiling, in particular DNA microarray analysis, has been widely used in attempts to reveal the underlying mechanisms of many diseases, different developmental stages, cellular responses to different conditions, and many other biological phenomena (for example, [1-3]). Gene expression signatures consisting of tens to hundreds of genes have been associated with many important aspects of the systems studied. To help realize the full potential of gene expression studies, a variety of methods, such as GenMAPP [4], GoMiner [5], DAVID [6] and its desktop version EASE [7], Catmap [8], ArrayXPath [9], and Gene Set Enrichment Analysis (GSEA) [10], have been developed to relate gene expression profiles or signatures to a broad range of biological categories. Although some of these methods include signaling pathways in their categories, their focus has not been on regulatory mechanisms that control the observed gene expression changes.

Signal transduction is at the core of many regulatory systems. Cellular functions such as growth, proliferation, differentiation, and apoptosis are regulated by signaling pathways. Appropriate regulation of such pathways is essential for the normal functioning of cells. Cells affected by disease often have one or several signaling pathways abnormally activated or inactivated. For example, cancer is a disease of deregulated cell proliferation and death [11]. To uncover mechanisms underlying cellular phenotypes, therefore, it is crucial to systematically analyze gene expression signatures in the context of signaling pathways. In signal transduction, ligands, usually from outside the cell, interact with receptors on the surface of the cell membrane or with nuclear receptors. These interactions trigger a cascade of biochemical reactions. Proteins called transcription factors (TFs) and cofactors are eventually transported to, or activated in, the nucleus of the cell where they turn transcription of target genes on or off. A signaling pathway is composed of a set of molecular components conveying the signal, such as ligands, receptors, enzymes, TFs, and cofactors.

When a pathway is activated, the expression levels of the components of the pathway are not necessarily affected. For example, mutation of a TF can change the expression levels of its target genes, without necessarily affecting the expression levels of the TF itself or other components of the pathway. Also, pathway components might not be regulated at the transcriptional level; instead, they are often regulated post-translationally, for example, by phosphorylation. Proteomic data could be used to detect such modifications and be used for pathway analysis, but currently there is a lack of such genome-wide protein data. It has beenpointed out that gene expression signatures may be more reliable indicators of pathway activities than protein data for single components in signaling pathways [12]. Taking all these considerations into account, we reason that the activity of a signaling pathway may currently be best characterized by the expression levels of its target genes. In support of this hypothesis, Breslin *et al*. [13] have shown the capacity of expression levels of known target genes to reflect pathway activities. However, knowledge about target genes of TFs is far from complete, which hampers accurate prediction of pathway activities. On the other hand, the *cis*-regulatory motifs to which TFs bind are often better characterized. For organisms with sequenced genomes, these motifs enable genome-wide identification of putative target genes by looking for potential TF binding sites in promoter sequences. Therefore, integrating regulatory motif analysis with pathway information would be a potential approach to break this bottleneck for pathway analysis. Recently, the feasibility ofusing putative binding sites to identify TFs responsible for gene expression signatures of human cancer has been demonstrated [14].

**Figure 1:**
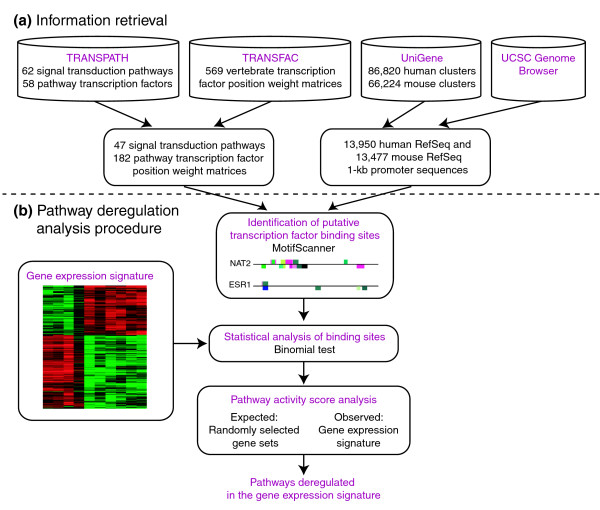
Overview of the method used to reveal pathways deregulated in gene expression signatures. **(a) **Information was retrieved and integrated from four sources: TRANSPATH, TRANSFAC, UniGene, and the UCSC Genome Browser. **(b) **Putative TF binding sites in promoter regions were identified using MotifScanner. Enrichment of putative transcription binding sites among genes in a gene signature was assessed using a binomial test. Each pathway was scored in terms of an enrichment for putative binding sites for the TFs mediating the pathway. The significance of a pathway's relevance for a gene signature was assessed by using randomly selected gene sets from the genome.

Here we present a strategy to discover activated and inactivated signaling pathways from gene expression signatures by using regulatory motif analysis (Figure 1). To achieve this goal, we began by extracting all signaling pathways in the TRANSPATH database [15], and characterized each pathway by the TFs that mediate it. In all human and mouse promoter sequences, we identified putative binding sites of all the TFs mediating pathways using TF binding site position weight matrices from the TRANSFAC database [16]. Next, we investigated promoters of genes in gene expression signatures for an enrichment of these putative binding sites. Finally, we measured the activity of a pathway in a gene expression signature in terms of the enrichment of binding motifs for the TFs mediating the pathway. Although the use of putative TF binding sites will introduce false-positive target genes for each TF, when the promoters of a set of co-expressed genes are enriched for a putative TF binding site, the gene set is also likely enriched for true target genes. Moreover, our strategy to integrate regulatory motif analysis with knowledge about which TFs act together in pathways further reduces the influence of false-positive targets on the identification of pathways.

Our results for six human and mouse gene expression signatures demonstrate the power of our method to identify relevant pathways. We compared our results with those obtained using two widely used methods for relating gene expression profiles to biological categories, EASE [7] and GSEA [10]. For data sets with known pathways activated, we found that our strategy identified the expected pathways whereas EASE and GSEA did not. Hence, our strategy provides additional information complementary to what can be obtained using current methods for biological interpretation of gene expression data.

## Results and discussion

### Gene signatures for oncogenic pathways

To examine the ability of our method to accurately detect the activity of pathways, we obtained gene signatures for three oncogenic pathways produced by Bild *et al*. [17]. These signatures consist of genes for which the expression levels in human mammary epithelial cells were highly correlated with the activation status of the oncogenes encoding E2F3 (268 genes), Myc (218 genes), or Ras (304 genes), respectively. These three oncogenic pathways are often activated in solid tumors, including breast tumors, where they contribute to tumor development or progression. Bild *et al*. verified the activation status of each pathway using various biochemical measurements and demonstrated that the expression patterns in each signature were specific to each pathway. Hence, these signatures are ideal for evaluating our strategy to identify activated pathways. The statistically significant pathways identified by our method for the three gene signatures are shown in Table 1.

**Table 1:** Significant pathways for oncogenic gene signatures

Pathway	TFs	Significant TFs	*P *value
**E2F3 gene signature**			
E2F	DP-1, E2F, p53	DP-1, E2F	<0.001
Caspases	CREB, Max, SRF, p53, AP-2α	AP-2α	<0.001
**Myc gene signature**			
AhR	AhR, ER-α, Sp1, p300, NF-κB, Arnt	AhR, Sp1, NF-κB, Arnt	<0.001
HIF-1	p53, p300, HIF-1α, HNF-4α2, Arnt	HIF-1α, Arnt	<0.001
Notch	Max, LEF-1, p300, c-Myc	Max, c-Myc	<0.001
EGF	c-Fos, Elk-1, Sp1, STAT3, c-Jun, STAT1α, c-Myc	Sp1, c-Myc	0.002
Caspases	CREB, Max, SRF, p53, AP-2α	Max, AP-2α	0.002
c-Kit	MITF, Sp1, Tal-1, p300, GATA-1	MITF, Sp1, Tal-1	0.006
**Ras gene signature**			
AhR	AhR, ER-α, Sp1, p300, NF-κB, Arnt	Sp1, NF-κB	<0.001
Apoptosis	p53, FOXO3a, NF-κB	p53, NF-κB	0.001
Caspases	CREB, Max, SRF, p53, AP-2α	CREB, p53, AP-2α	0.004
RANK	MITF, PU.1, c-Jun, NF-κB	PU.1, NF-κB	0.008
TNFα	AP-1, NF-κB	AP-1, NF-κB	0.009
TLR4	CREB, CRE-BP2, STAT1, Elk-1, p300, IRF-3, IRF-7, NF-κB	CREB, CRE-BP2, NF-κB	0.015
MAPK	CREB, Elk-1, p53, c-Jun, c-Myc	CREB, p53	0.023
TLR3	CRE-BP2, p300, c-Jun, IRF-3, IRF-7, NF-κB	CRE-BP2, NF-κB	0.034
p38	ELk-1, p53, MITF, PPAR-α, CHOP-10, Max, CREB, PU.1, MRF4, HNF-1α, CRE-BP2, NF-AT2, STAT3	p53, PPAR-α, CHOP-10, CREB, PU.1, CRE-BP2	0.035
Stress	PPAR-γ, c-Ets-1, PPAR-α, Max, NF-AT2, HSF1, c-Jun, Elk-1, p53, CHOP-10, CREB, CRE-BP2, RXR-α, HNF-1α, STAT3, MRF4	PPAR-α, p53, CHOP-10, CREB, CRE-BP2	0.037

The E2F pathway was extremely significant for the E2F3 gene signature. E2F3 is a member of the E2F TF family (E2Fs). E2Fs can induce cell cycle G1 to S transition and activate many genes encoding proteins essential for DNA replication [18,19]. E2F1, another member of the E2Fs, can form dimers with DP-1, making this activation more efficient [20]. Our method identified both E2F1 (*P *< 0.001) and DP-1 (*P *< 0.001) as significant TFs for this signature.

TRANSPATH does not contain a strictly defined Myc pathway, but it includes three pathways containing c-Myc as a TF: the epidermal growth factor (EGF), Notch, and mitogen-activated protein kinase (MAPK) pathways. We identified c-Myc as a significant TF for this signature (*P *< 0.001), and both the EGF and the Notch pathways were found to be significant. The MAPK pathway was not found to be significant. The only significant TF found for the MAPK pathway was c-Myc, perhaps suggesting that induction of c-Myc is not sufficient to deregulate this pathway. Consistent with this suggestion, it has been shown that elevated c-Myc expression is not sufficient for tumorigenesis in human mammary epithelial cells [21]. Interestingly, we also found the hypoxia-inducible pathway HIF-1 significant. Studies have shown that HIF-1 is activated in many tumors, including breast cancer [22], as a consequence of a shortage in oxygen supply during sustained tumor growth. Moreover, it has been reported that HIF-1α counteracts Myc to induce cell cycle arrest, and HIF-1α down-regulates Myc-activated genes [23].

In the analysis of the Ras gene signature, we found the MAPK and p38 pathways to be significantly relevant. This finding is consistent with the fact that Ras activates MAPKs, including ERK and p38. It has been shown in human fibroblasts that a sustained high intensity Ras signal induces increased expression of MEK and ERK, eventually resulting in stimulation of the p38 pathway [24] and that the p38 pathway provides negative feedback for Ras proliferation [25]. Several of the pathways we found to be significant contained nuclear factor (NF)-κB as a significant TF (*P *= 0.002), including the receptor activator of NF-κB (RANK) and tumor necrosis factor-α pathways. It has been shown that NF-κB has an essential role in breast cancer progression, and activation of NF-κB signaling is especially required for the epithelial-mesenchymal transition in Ras-transformed epithelial cells [26]. We identified the stress pathway as affected, perhaps only because this pathway overlaps the p38 pathway. Also, we identified the TLR3 and TLR4 pathways as responsive to Ras stimulation. A recent study has shown that toll-like receptors (TLRs) are expressed in a variety of tumors and trigger tumor self-protection mechanisms [27], making it plausible that they are induced by Ras activation.

In addition to those pathways affected specifically for an oncogenic activation signature, the caspases pathway was found to be significantly affected for all three signatures. The caspases pathway triggers cell death. Because evasion of cell death is essential for tumor development [11], it is likely that this pathway is repressed regardless of which of the oncogenes is activated. Indeed, it has been indicated that over-expression of E2F3 or Ras induces tumor invasion through interaction with AP-2α, a characteristic TF in the caspases pathway, in epithelial cells of bladder cancer [28]. It has also been shown that c-Myc represses AP-2α *trans*-activation [29]. Another pathway found to be affected for more than one signature was the AhR pathway, which was found to be significant for both the Myc and Ras gene signatures. It has been demonstrated that the AhR TF is constitutively active at high levels in mammary tumors compared to in normal mammary glands, suggesting that it contributes to ongoing mammary tumor cell growth [30]. For all identified significant pathways, a total of 19 significant TFs were found. Of these, only AP-2α was significant for all three signatures and only AhR, Sp1, and NF-κB were significant for two signatures. These small overlaps show that we do not find the same set of TFs for each signature and verify the conclusion of Bild *et al*. [17] that the signatures are specific to each pathway. Taken together, our results for these three oncogenic gene signatures demonstrate the power of our method to accurately identify the known active pathways. Moreover, we found additional pathways known to be relevant for each oncogenic pathway. These results highlight the potential of our method to generate hypotheses for connections between pathways.

We also looked into pathway activities for each oncogenic signature by analyzing the up-regulated and down-regulated genes separately. Each oncogenic signature was divided into two signatures, one containing the up-regulated and one containing the down-regulated genes. For the up-regulated signatures we obtained essentially the same result as for theoriginal signatures containing both up- and down-regulated genes. In contrast, very few significant pathways were found for the down-regulated signatures, likely because these signatures contained very few genes. For example, there were only 32 genes found to be down-regulated by E2F3. Suchsmall numbers do not allow for a detailed analysis of whether our method would benefit from analyzing up- and down-regulated genes separately. In the following analysis, we have used signatures containing both up- and down-regulated genes for our method.

### Gene signatures for the TGF-β pathway

Sets of genes claimed to belong to a gene signature are often sensitive tosample selection and have small overlaps in different studies [31,32]. This issue has raised debate about the credibility of such signatures. A possible explanation for small overlaps is that there may be redundancy in expression profiles; many gene sets are equally good at distinguishing a phenotype of interest. In this case, gene sets with small overlaps may still arise from activation or repression of identical pathways.

To validate our method as a guide to pathway analysis in this regard, we analyzed target genes of the transforming growth factor (TGF)-β pathway from two independent studies. One data set contains 360 genes identified by comparing expression profiles of murine embryonic fibroblast(MEF) cells deficient in Smad2, Smad3, or MAPK ERK, which are mediators ofTGF-β signaling, with those of wild-type MEFs in response to 1, 2, or 4 hours of TGF-β stimulation [33]. The other data set contains 465 targets differentially expressed between MEFs with the TGF-β receptor Alk5 knocked out and wild-type MEFs stimulated with TGF-β for 2, 4, or 16 hours [34].

Whereas there are only 29 genes in common for the two data sets, manyof the active pathways we found are the same (Table 2). In particular, all five pathways with *P *< 0.001 for the Karlsson *et al*. [34] data set also have *P *< 0.001 for the Yang *et al*. [33] data set. We identified the TGF-β pathway as significant for the Yang *et al*. target genes, but not forthe Karlsson *et al*. genes. This discrepancy is possibly due to the different durations of TGF-β stimulation in the two experiments. Yang *at al*. reported that Smad3/Smad4 binding motifs are present only in immediate-early target genes but not in the intermediate ones [33]. The lack of an overabundance of genes containing Smad binding motifs in the Karlsson *et al*. data set suggests that it consists of intermediate or late response genes. A targetgene of TGF-β signaling is Myc and it is one of the genes in commonfor both data sets. The repression of Myc by TGF-β stimulation is mediated by the TFs E2F4/5 and DP-1 [35]. In agreement with this picture, we found all six pathways that were significant for the Myc gene signature (Table 1) as well as the E2F pathway to be significant for both TGF-β data sets (Table 2).

**Table 2:** Significant pathways for TGF-β gene signatures

Pathway	TFs	Significant TFs	*P *value
**Yang *et al*. gene signature**			
AhR	AhR, ER-α, Sp1, p300, NF-κB, Arnt	AhR, Sp1, p300, NF-κB, Arnt	<0.001
EGF	c-Fos, Elk-1, Sp1, STAT3, c-Jun, STAT1α, c-Myc	Sp1, c-Jun, c-Myc	<0.001
c-Kit	MITF, Sp1, Tal-1, p300, GATA-1	Sp1, p300	<0.001
p53	TFIIA, E2F1, p53, p300, BRCA1, YY1	E2F1, p53, p300, BRCA1	<0.001
Caspases	CREB, Max, SRF, p53, AP-2α	CREB, Max, p53, AP-2α	<0.001
MAPK	CREB, Elk-1, p53, c-Jun, c-Myc	CREB, p53, c-Jun, c-Myc	<0.001
E2F	DP-1, E2F, p53	DP-1, E2F, p53	<0.001
HIF-1	p53, p300, HIF-1α, HNF-4α2, Arnt	p53, p300, HIF-1α, Arnt	<0.001
Stress	PPAR-γ, c-Ets-1, PPAR-α, Max NF-AT2, HSF1, c-Jun, Elk-1, p53, CHOP-10, CREB, CRE-BP2, RXR-α, HNF-1α, STAT3, MRF4	Max, c-Jun, p53, CREB, CRE-BP2, RXR-α	0.001
TLR3	CRE-BP2, p300, c-Jun, IRF-3, IRF-7, NF-κB	CRE-BP2, p300, c-Jun, NF-κB	0.002
TLR4	CREB, CRE-BP2, STAT1, Elk-1, p300, IRF-3, IRF-7, NF-κB	CREB, CRE-BP2, p300, NF-κB	0.002
p38	ELk-1, p53, MITF, PPAR-α, CHOP-10, Max, CREB, PU.1, HNF-1α, CRE-BP2, NF-AT2, STAT3, MRF4	p53, Max, CREB, CRE-BP2	0.003
JNK	CRE-BP2, p53, HSF1, PPAR-γ, STAT3, c-Jun, c-Ets-1	CRE-BP2, p53, c-Jun	0.004
TGF-β	LEF-1, CRE-BP2, Smad2, Smad3, Smad4	CRE-BP2, Smad4	0.006
EDAR	c-Jun, NF-κB	c-Jun, NF-κB	0.015
IL-1	ELk-1, c-Jun, NF-κB	c-Jun, NF-κB	0.015
TCR2	c-Jun, NF-κB, NF-AT	c-Jun, NF-κB	0.018
RANK	MITF, PU.1, c-Jun, NF-κB	c-Jun, NF-κB	0.020
Hypoxia	ER-α, p53, AP-1, HIF-1α	p53, HIF-1α	0.033
Notch	Max, LEF-1, p300, c-Myc	Max, p300, c-Myc	0.037
**Karlsson *et al*. gene signature**			
AhR	AhR, ER-α, Sp1, p300, NF-κB, Arnt	AhR, Sp1, Arnt	<0.001
EGF	c-Fos, Elk-1, Sp1, STAT3, c-Jun, STAT1α, c-Myc	Sp1, STAT1α, c-Myc	<0.001
c-Kit	MITF, Sp1, Tal-1, p300, GATA-1	Sp1, Tal-1	<0.001
p53	TFIIA, E2F1, p53, p300, BRCA1, YY1	E2F1, BRCA1	<0.001
Caspases	CREB, Max, SRF, p53, AP-2α	Max, AP-2α	<0.001
E2F	DP-1, E2F, p53	DP-1, E2F	0.002
HIF-1	p53, p300, HIF-1α, HNF-4α2, Arnt	HIF-1α, Arnt	0.006
Notch	Max, LEF-1, p300, c-Myc	Max, c-Myc	0.019

The fibroblasts used by Yang *et al*. to identify TGF-β responsive genes included MEFs with genetic ablation of MAPK ERK. The oncogene Ras activates ERK, and eight of the ten pathways we found to be significant for the Ras gene signature (Table 1) were also found to be significant for the Yang *et al*. gene signature (Table 2). This finding indicates that the Yang *et al *gene signature is a mixture of the transcriptional response to both MAPK and Smad signaling. For this data set, four pathways appeared as significant only because they contain the TFs c-Jun and NF-κB. These two TFs also appear in other significant pathways supported by additional significant TFs, including the AhR, EGF, MAPK, and p38 pathways. Biochemical investigations are required to reveal if the pathways with only c-Jun and NF-κB are indeed deregulated, or if they are false positives likely to go away as the information in pathway databases improves.

This analysis of TGF-β signaling provides a demonstration that pathway analysis can be used to find common pathways underlying gene sets with small overlaps. In addition, we have again verified that our method identifies relevant pathways.

### Poor prognosis gene signature for breast cancer

Finally, we tested the ability of our method to identify signaling pathways involved in a disease by using a gene expression signature from breast tumor samples. We used a signature distinguishing patients who developed distant metastases within five years from patients who remained disease free for at least five years [36]. This poor prognosis gene signature contains 70 genes that we investigated for pathway activities. The signature consists of genes annotated as being involved in cell cycle, invasion, metastasis, and angiogenesis [36].

Consistent with the functional annotation of the genes, we found that the E2F pathway, a pathway that regulates the cell cycle, was most significantly associated with the poor prognosis signature (Table 3). Activation of the E2F pathway can induce the transition from G1 to S phase in the cell cycle. The percentage of cells in a tumor cell population that are in S phase is known to be associated with shorter disease-free survival [37]. We also found the AhR pathway to be significant (Table 3). The AhR pathway has been suggested to inhibit apoptosis while promoting transition to an invasive, metastatic phenotype for breast tumors [30]. Interestingly, we found the caspases pathway, which regulates apoptosis, to be significant (Table 3). This finding is consistent with the indication in recent studies that apoptosis is a central mechanism regulating metastasis [38]. We note that the pathways found are similar to those significant for the E2F3 oncogenicgene signature (Table 1), suggesting that the poor prognosis signature largely reflects cell proliferation. Our analysis of the poor prognosis signature highlights the potential of our method to reveal pathways that both are consistent with functional annotations of genes in signatures andprovide a more detailed insight into the molecular mechanisms underlying the annotations.

**Table 3:** Significant pathways for the breast cancer prognosis gene signature

Pathway	TFs	Significant TFs	P value
E2F	DP-1, E2F, p53	DP-1, E2F	<0.001
AhR	AhR, ER-α, Sp1, p300, NF-κB, Arnt	AhR, Sp1	0.017
Caspases	CREB, Max, SRF, p53, AP-2α	AP-2α	0.039

### Comparison with EASE and GSEA for oncogenic pathway profiles

We compared our method with methods that relate gene expression signaturesor profiles to gene annotations. Two widely used methods for such analysisare EASE [7] and GSEA [10]. EASE uses a gene signature and can, among other things, search for an enrichment in the signature of genes annotated as components of pathways in the KEGG, GenMAPP, and BBID pathway databases. GSEA uses entire gene expression profiles to evaluate whether a pre-defined set of genes shows statistically significant, concordant differences between two biological states. GSEA provides a collection of gene sets called the Molecular Signature Database (MSigDB), which contains two collections of gene sets relevant for pathway analysis. The gene set C2 (curated gene sets) includes sets of pathway genes from the BioCarta, GenMAPP, and Signal transduction knowledge environment (STKE) databases, but also numerous published gene signatures [10]. The gene set C3 (motif gene sets) includes sets of genes annotated as TF targets using TRANSFAC [39]. Given the differences between these two methods, we think a comparison with EASE and GSEA will highlight important differences between our method and methods that identify pathways based on pathway components. We used the three oncogenic pathway data sets for this comparison because they are ideal to evaluate whether pathway activation can be identified from gene expression profiles since each data set reflects activation of a known pathway.

#### EASE results

For the E2F3 signature, EASE identified a few cell cycle-related pathways as significant (EASE score <0.05): 'Cell cycle' and 'Cell growth and death' from KEGG, 'Cell cycle' from GenMAPP, as well as 'RBphosphoE2F' and 'cyclin-CDK complexes' from BBID. They were all identified by a set of cell cycle genes. In addition, 'Purine metabolism' from KEGG and 'Wnt signaling' from GenMAPP were found to be significant. All of these pathways reflect downstream effects of E2F3 activation. However, the 'E2F transcriptional activity cell cycle' pathway from BBID was not found to be significant at all (EASE score of 1.0). For the Myc signature, EASE identified 'Fructose and mannose metabolism' and 'Carbohydrate metabolism' from KEGG as well as 'Glycolysis and gluconeogenesis' from GenMAPP as significant pathways (EASE score <0.05). These three pathways were essentially identified by the same genes. In contrast, pathways with Myc itself as a component, including the 'Myc network' and 'G1-phase transition by Myc' from BBID, were found to be insignificant (all had EASE scores of 1.0). For the Ras signature, two pathways from KEGG, 'Signal transduction' and 'Phosphatidylinositol signaling system', were found to be significant (EASE score <0.05). It has been indicated that Ras activates the phosphatidylinositol signaling system, although not at levels sufficient for oncogenic transformation of human mammary epithelial cells [21]. However, the pathways 'MAPK signaling' from KEGG (EASE score = 0.35) and 'MAPK cascade' from GenMAPP (EASE score = 1.0) were not significant. For each signature, we also analyzed the up- and down-regulated genes separately. We found the results for signatures consisting of up-regulated genes to be almost identical to the results obtained using the total signatures, while very few significant pathways were found for the down-regulated genes. Together, these results for gene signatures of active oncogenic pathways suggest that EASE identifies downstream effects but not the known activated pathways.

#### GSEA results

We submitted the expression profiles for each oncogenic pathway to GSEA and searched for enriched gene sets among the C2 gene set collection from MSigDB. We used default settings for GSEA, which means that up- and down-regulated genes were analyzed separately. Surprisingly, for the E2F3 and Ras data, no gene sets were found to be significant (false discovery rate (FDR) < 25%). For the E2F3 data, none of the gene sets related to E2F obtained a *P *value below 0.18. For the Ras data, the RAS pathway from BioCarta obtained a *P *value of 0.32 and none of five MAPK pathways obtained *P *values below 0.05. A gene set described as genes of the MAPK cascade, with no further information, obtained a *P *value of 0.027 but was only ranked as gene set 59. For the Myc data, no significant gene sets were found for genes up-regulated by Myc activation (FDR < 25%). However, five of the ten top ranked gene sets were related to Myc. Four sets consisted of genes found by other gene expression profiling studies to be up-regulated by Myc and one set was a database of identified direct targets of Myc. On the other hand, there were 393 gene sets significant for genes down-regulated by Myc (FDR < 25%), but no Myc-related gene set obtained a *P *value below 0.05. We also analyzed the data sets with GSEA such that up- and down-regulated genes were not separated and obtained gene sets ranked essentially in the same order as for up-regulation separately. However, for this analysis, GSEA identified 855, 966, and 829 sets significant at a FDR < 25% out of 1,287 gene sets for the E2F3, Myc, and Ras data, respectively, indicating that the significance calculations in GSEA are highly sensitive to changes in parameter settings. These results reinforce that the genes for which expression correlated with activation of oncogenic pathways are the target genes of the oncogenic pathways rather than the components of the pathways.

We also ran GSEA for the oncogenic profiles using the C3 gene set collection from MSigDB to search for TFs potentially regulating the gene expression profiles. For the E2F3 data, 568 gene sets were significant at a FDR < 25%. Of the ten top ranked motifs, eight were binding motifs for TFs in the E2F family. For the Myc data, GSEA identified eight gene sets at a FDR < 25%, including binding motifs for Myc and Nmyc. No significant gene sets were found for the Ras data. We also performed this motif analysis for up- and down-regulated genes together. Again, we obtained gene sets ranked in similar order as for the up-regulated genes analyzed separately, but with the majority of all gene sets significant at a FDR < 25%.

#### The methods provide complementary information

Our comparison with EASE and GSEA has shown that identifying pathway deregulation from gene expression profiles by mapping genes to pathway components is difficult. Instead, we find, using both Toucan [40,41] as a part of our strategy and GSEA with the C3 (motif) gene sets, that characteristically expressed genes are more likely target genes of the deregulated pathways. With this in mind, it is not surprising that our strategy was better than EASE and GSEA at identifying the expected activated pathways for the oncogenic pathway profiles. On the other hand, by having the potential to identify downstream effects of the deregulated pathways, EASE may provide information complementary to our method. Although mapping to gene sets consisting of pathway components using GSEA did not identify the deregulated pathways, GSEA can be used with a variety of other gene sets that can provide valuable information. Our GSEA results for the Myc data show that gene sets based on gene expression signatures from pathway characterization experiments can be used to identify pathway deregulation in other gene expression data sets. Such signatures are likely a mixture of direct targets and genes affected downstream. Motif analysis, as part of our strategy, has the advantage of emphasizing target genes, which allows for more accurate identification of signaling pathway deregulation. Our GSEA results for the C3 (motif) gene sets also show that GSEA is useful for identifying TFs whose deregulation results in an observed gene expression profile. However, our results indicate that the significance statistics that Toucan uses are more robust for the discovery of significant binding motifs. In addition, the results obtained with our method suggest that a gene set for a pathway could be generated by merging all motif gene sets for the TFs involved in the pathway. Such pathway gene sets could be very useful for GSEA analysis.

## Conclusion

We present a strategy to identify signaling pathways whose deregulation results in an observed gene expression signature. The strategy is based on combining identification of putative TF binding sites in promoter regions of genes with knowledge about which TFs act in the same pathway. The major conclusions from our results for six human and mouse gene expression signatures are as follows. First, it is feasible to identify pathways deregulated in mammalian gene expression signatures by viewing such signatures as a collection of target genes of the TFs mediating the pathways. Second, while binding site analysis alone can identify key TFs, combining such analysis with pathway information improves the potential to direct attention to possible mechanisms driving an observed transcriptional response. Third, mapping gene expression signatures onto pathways by motif analysis can guide the identification of common regulatory programs driving different signatures with small overlaps, as well as the identification of diverse regulatory programs driving a single signature. Moreover, our strategy provides information complementary to widely used methods for biological interpretation of gene expression data such as EASE and GSEA. While such methods, for example, can verify the biological consistency of gene expression data to pathway signatures in the literature, we found that our strategy was better at identifying the pathways known to be deregulated for many of the data sets. As pathway databases are steadily growing in size and quality, we expect that methods combining regulatory motif analysis with pathway information will be even more useful in the future.

## Materials and methods

### Pathway information retrieval

Signal transduction pathways were taken from the TRANSPATH database (release 7.1). For the 62 pathways defined in the database, 58 components were identified as TFs mediating at least one pathway. We extracted pathway-TF pairs from the map files provided by TRANSPATH and extracted DNA binding motifs of these TFs from TRANSFAC (release 10.1). The binding motifs used were 6-24 bp long and each was represented by a position-weight-matrix (PWM) that indicates the experimentally determined frequency of the four nucleotides at each position. Some TFs have multiple DNA binding motifs, and each binding motif is associated with one PWM. The 58 pathway TFs were associated with 182 PWMs. There were 47 pathways represented by at least one PWM. These 47 pathways were used in our subsequent analysis (Figure 1a).

### Identification of transcription factor binding sites

Each human and mouse cluster in the UniGene database (human build 193; mouse build 155) was associated with RefSeq reference sequences using ACID [42]. Clusters that did not match a RefSeq or matched multiple RefSeqs were excluded from the analysis. This procedure resulted in 13,950 human and 13,477 mouse RefSeqs for which we retrieved 1 kb promoter sequences from the University of California Santa Cruz Genome Browser [43] using human assembly hg18 and mouse assembly mm7 (Figure 1a). Putative TF binding sites in the promoter sequences were identified by using MotifScanner, a part of the Toucan software [40,41], which can search for the occurrences of a list of known motifs in each query sequence. MotifScanner requires several arguments including: a set of query sequences; a background model that scores the frequencies of single nucleotides or oligonucleotides of fixed size; and a set of motifs represented by PWMs. In our analysis, all 1 kb promoter sequences for a species were used both as a query set and to generate a background model for oligonucleotides of size three [44]. All PWMs for the pathway TFs were used when searching for putative binding sites. Default values were used for all other MotifScanner parameters. For each promoter sequence, MotifScanner outputs the number of occurrences for each motif.

### Statistical analysis of binding sites

The genome-wide frequency (*f*) of each motif (*m*) is calculated by dividing the observed number of occurrences (*K*) of this motif in all human or mouse promoter sequences (*N*) with the number of possible start positions *R*(*N*):

fm=KR(N).
 MathType@MTEF@5@5@+=feaafiart1ev1aqatCvAUfeBSjuyZL2yd9gzLbvyNv2Caerbhv2BYDwAHbqedmvETj2BSbqee0evGueE0jxyaibaiKI8=vI8tuQ8FMI8Gi=hEeeu0xXdbba9frFj0=OqFfea0dXdd9vqai=hGuQ8kuc9pgc9s8qqaq=dirpe0xb9q8qiLsFr0=vr0=vr0dc8meaabaqaciGacaGaaeqabaqadeqadaaakeaacaWGMbWaaSbaaSqaaiaad2gaaeqaaOGaeyypa0ZaaSaaaeaacaWGlbaabaGaamOuaiaacIcacaWGobGaaiykaaaacaGGUaaaaa@3ADC@

The possible number of start positions (*R*) in *n *promoter sequences for a motif was approximated as:

R(n)=2×∑i=1n(Li−w+1),
 MathType@MTEF@5@5@+=feaafiart1ev1aqatCvAUfeBSjuyZL2yd9gzLbvyNv2Caerbhv2BYDwAHbqedmvETj2BSbqee0evGueE0jxyaibaiKI8=vI8tuQ8FMI8Gi=hEeeu0xXdbba9frFj0=OqFfea0dXdd9vqai=hGuQ8kuc9pgc9s8qqaq=dirpe0xb9q8qiLsFr0=vr0=vr0dc8meaabaqaciGacaGaaeqabaqadeqadaaakeaacaWGsbGaaiikaiaad6gacaGGPaGaeyypa0JaaGOmaiabgEna0oaaqahabaGaaiikaiaadYeadaWgaaWcbaGaamyAaaqabaaabaGaamyAaiabg2da9iaaigdaaeaacaWGUbaaniabggHiLdGccqGHsislcaWG3bGaey4kaSIaaGymaiaacMcacaGGSaaaaa@4787@

where *Li *is the length of the *i*th sequence and *w *is the length of the motif. The *P *value of observing *k *or more occurrences of the motif *m *in *n *(*n *≤ *N*) promoter sequences is calculated by a binomial test (Figure 1b) as described in [40]:

P−value(m)=∑j=kR(n)(R(n)j)×fmj×(1−fm)R(n)−j.
 MathType@MTEF@5@5@+=feaafiart1ev1aaatCvAUfeBSjuyZL2yd9gzLbvyNv2Caerbhv2BYDwAHbqedmvETj2BSbqee0evGueE0jxyaibaiKI8=vI8tuQ8FMI8Gi=hEeeu0xXdbba9frFj0=OqFfea0dXdd9vqai=hGuQ8kuc9pgc9s8qqaq=dirpe0xb9q8qiLsFr0=vr0=vr0dc8meaabaqaciGacaGaaeqabaqadeqadaaakeaacaWGqbGaeyOeI0ccbaGaa8NDaiaa=fgacaWFSbGaa8xDaiaa=vgacaGGOaGaamyBaiaacMcacqGH9aqpdaaeWbqaamaabmaabaqbaeqabiqaaaqaaiaadkfacaGGOaGaamOBaiaacMcaaeaacaWGQbaaaaGaayjkaiaawMcaaiabgEna0kaadAgadaqhaaWcbaGaamyBaaqaaiaadQgaaaaabaGaamOAaiabg2da9iaadUgaaeaacaWGsbGaaiikaiaad6gacaGGPaaaniabggHiLdGccqGHxdaTcaGGOaGaaGymaiabgkHiTiaadAgadaWgaaWcbaGaamyBaaqabaGccaGGPaWaaWbaaSqabeaacaWGsbGaaiikaiaad6gacaGGPaGaeyOeI0IaamOAaaaakiaac6caaaa@5CFF@

Thus, a small *P *value indicates an enrichment for motif *m *in the promoters of genes in a gene signature.

### Statistical analysis of pathway activities

The activity of a pathway in a gene expression signature was assessed by the enrichment of the binding motifs for the TFs mediating this pathway (Figure 1b). Letting TF(*p*) denote the set of TFs for a pathway *p*, and M(*t*) the set of binding motifs for a TF *t*, we used the *P *values for the motifs (equation 3) to first define a score for a TF *t *as:

S(t)=−∑m∈M(t)log(P−value(m)),
 MathType@MTEF@5@5@+=feaafiart1ev1aqatCvAUfeBSjuyZL2yd9gzLbvyNv2Caerbhv2BYDwAHbqedmvETj2BSbqee0evGueE0jxyaibaiKI8=vI8tuQ8FMI8Gi=hEeeu0xXdbba9frFj0=OqFfea0dXdd9vqai=hGuQ8kuc9pgc9s8qqaq=dirpe0xb9q8qiLsFr0=vr0=vr0dc8meaabaqaciGacaGaaeqabaqadeqadaaakeaacaWGtbGaaiikaiaadshacaGGPaGaeyypa0JaeyOeI0YaaabuaeaaieaacaWFSbGaa83Baiaa=DgacaGGOaGaa8huaGGaaiab+jHiTerbuLwBLnhiov2DGi1BTfMBaGabaiaa9zhacaqFHbGaa0hBaiaa9vhacaqFLbGaaiikaiaad2gacaGGPaGaaiykaiaacYcaaSqaaiaad2gacqGHiiIZcaWFnbGaaiikaiaadshacaGGPaaabeqdcqGHris5aaaa@5309@

and second a score for a pathway *p *as:

S(p)=∑t∈TF(p)S(t).
 MathType@MTEF@5@5@+=feaafiart1ev1aqatCvAUfeBSjuyZL2yd9gzLbvyNv2Caerbhv2BYDwAHbqedmvETj2BSbqee0evGueE0jxyaibaiKI8=vI8tuQ8FMI8Gi=hEeeu0xXdbba9frFj0=OqFfea0dXdd9vqai=hGuQ8kuc9pgc9s8qqaq=dirpe0xb9q8qiLsFr0=vr0=vr0dc8meaabaqaciGacaGaaeqabaqadeqadaaakeaacaWGtbGaaiikaiaadchacaGGPaGaeyypa0ZaaabuaeaacaWGtbGaaiikaiaadshacaGGPaaaleaacaWG0bGaeyicI4mcbaGaa8hvaiaa=zeacaGGOaGaamiCaiaacMcaaeqaniabggHiLdGccaGGUaaaaa@43D3@

We generated gene sets of the same size as the gene signature by randomly selecting genes from the human or mouse genome. We calculated a *P *value for pathway *p *by comparing *S*(*p*) with scores obtained using these randomly selected gene sets. A *P *value for TF *t *was calculated as for pathway *p *but using the TF score *S*(*t*) instead of the pathway score. In this way two types of *P *values are obtained: one for TFs and one for pathways. We used 1,000 randomly selected sets in each of our analyses. TFs with *P *< 0.1 were considered significant. Pathways were considered significant if they met two criteria: a pathway *P *value < 0.05; and at least two significant TFs or one significant TF unique for the pathway.

### EASE and GSEA analysis

In the EASE analysis, we selected the categories BBID pathway, GenMAPP pathway, and KEGG pathway, used the EASE score as the primary score, and used all mouse or human genes as the general population of genes. For all other EASE settings, we used default values. Pathways that obtained an EASE score smaller than 0.05 were considered significant.

We used default values for parameters in the GSEA analysis: genes were ranked according to how their expression levels correlate with phenotypes using the signal-to-noise ratio, and phenotype permutations were used for assessments of significance. A FDR maximum of 25% was used to identify significant gene sets as recommended by GSEA. When presenting results for specific gene sets nominal, uncorrected *P *values are shown. When analyzing up- and down-regulated genes together the absolute value of the signal-to-noise ratio was used to rank genes. Gene sets were obtained from MSigDB version 2 (January 2007 release).

### Gene signatures

We obtained six different publicly available human and mouse gene signatures. Gene identifiers were mapped to UniGene clusters using ACID [42]. Gene identifiers that mapped to multiple UniGene clusters were removed from further analysis.

### Availability

Software for the method was written using the PERL programming language and is freely available upon request.

## Additional data files

The following additional data are available with the online version of this paper. Additional data file 1 is a file in tab-delimited format listing the results for all pathways for the E2F3 gene signature. Additional data file 2 is a file in tab-delimited format listing the results for all pathways for the Myc gene signature. Additional data file 3 is a file in tab-delimited format listing the results for all pathways for the Ras gene signature. Additional data file 4 is a file in tab-delimited format listing the results for all pathways for the Yang *et al*. [33] gene signature. Additional data file 5 is a file in tab-delimited format listing the results for all pathways for the Karlsson *et al*. [34] gene signature. Additional data file 6 is a file in tab-delimited format listing the results for all pathways for the breast cancer prognosis gene signature.

## Supplementary Material

Additional data file 1 Results for all pathways for the E2F3 gene signature Click here for file

Additional data file 2 Results for all pathways for the Myc gene signature Click here for file

Additional data file 3 Results for all pathways for the Ras gene signature Click here for file

Additional data file 4 Results for all pathways for the Yang *et al*. [33] gene signature Click here for file

Additional data file 5 Results for all pathways for the Karlsson *et al*. [34] gene signature Click here for file

Additional data file 6 Results for all pathways for the breast cancer prognosis gene signature Click here for file
